# Presenilins as Drug Targets for Alzheimer’s Disease—Recent Insights from Cell Biology and Electrophysiology as Novel Opportunities in Drug Development

**DOI:** 10.3390/ijms19061621

**Published:** 2018-05-31

**Authors:** R. Scott Duncan, Bob Song, Peter Koulen

**Affiliations:** 1 Vision Research Center, Department of Ophthalmology, School of Medicine, University of Missouri-Kansas City, Kansas City, MO 64108, USA; duncanrs@umkc.edu (R.S.D.); bsong1993@gmail.com (B.S.); 2 Department of Biomedical Science, School of Medicine, University of Missouri-Kansas City, Kansas City, MO 64108, USA

**Keywords:** γ-secretase, amyloid beta, calcium signaling, drug target discovery, endoplasmic reticulum, inositol 1,4,5-trisphosphate receptor, ion channel, oxidative stress, ryanodine receptor, therapy

## Abstract

A major cause underlying familial Alzheimer’s disease (AD) are mutations in presenilin proteins, presenilin 1 (PS1) and presenilin 2 (PS2). Presenilins are components of the γ-secretase complex which, when mutated, can affect amyloid precursor protein (APP) processing to toxic forms of amyloid beta (Aβ). Consequently, presenilins have been the target of numerous and varied research efforts to develop therapeutic strategies for AD. The presenilin 1 gene harbors the largest number of AD-causing mutations resulting in the late onset familial form of AD. As a result, the majority of efforts for drug development focused on PS1 and Aβ. Soon after the discovery of the major involvement of PS1 and PS2 in γ-secretase activity, it became clear that neuronal signaling, particularly calcium ion (Ca^2+^) signaling, is regulated by presenilins and impacted by mutations in presenilin genes. Intracellular Ca^2+^ signaling not only controls the activity of neurons, but also gene expression patterns, structural functionality of the cytoskeleton, synaptic connectivity and viability. Here, we will briefly review the role of presenilins in γ-secretase activity, then focus on the regulation of Ca^2+^ signaling, oxidative stress, and cellular viability by presenilins within the context of AD and discuss the relevance of presenilins in AD drug development efforts.

## 1. Introduction

Presenilins have long been known to play a role in familial Alzheimer’s disease (AD) pathogenesis [1]. With two presenilin genes in vertebrates, homologs of the human genes *PSEN1* and *PSEN2*, the two resulting presenilin proteins, presenilin 1 (PS1) and presenilin 2 (PS2) [1] are constituents of the multi-subunit γ-secretase complex which facilitates proteolytic processing of amyloid precursor protein (APP) [2]. Mutations in APP lead to accumulation of amyloid-beta peptides (Aβ), which can be toxic to neural tissue and contribute to AD pathology in the brain [2] with recent studies indicating that the formation of annular protofibrils by Aβ leads to membrane permeabilization and subsequent dysregulation of ion homeostasis [3]. PS1, specifically, is associated with familial AD in part by influencing Ca^2+^ signaling [4], yet there is still much to be uncovered about presenilins with new studies revealing more about non-canonical (non-γ-secretase–related) functions. Here, we discuss the role of PS1 and PS2 in cellular oxidative stress, in protein degradation/autophagy, and in regulating intracellular endoplasmic reticulum (ER) Ca^2+^ channels (i.e., inositol 1,4,5-trisphosphate receptors (IP_3_Rs) and ryanodine receptors (RyRs)). Investigating this involvement of presenilins in Ca^2+^ signaling results in unique challenges due to the ubiquitous expression of IP_3_Rs and RyRs by a wide range of cell types in almost every tissue and organ. This challenge also represents a unique opportunity for drug target discovery and clinical drug development efforts by taking advantage of recently identified mechanisms that place presenilins at the crossroads of oxidative stress, calcium signaling, and neuronal viability. Combining such insights with the newly identified role of presenilin involvement in neuronal calcium signaling represents novel opportunities in drug development for Alzheimer’s disease, the focus of the present review.

## 2. γ-Secretase Activity of Presenilin

The role of presenilins, APP and γ-secretase in AD pathogenesis has been widely studied. Presenilin proteins, PS1 and PS2, are constituents of the γ-secretase complex, which carry out amyloid precursor protein (APP) proteolytic processing [2]. Three new novel PS1 mutations have been uncovered in patients with a vast heterogeneity of clinical phenotypes [5]. Investigation of wild-type γ-secretase with six familial Alzheimer’s disease (FAD) mutants in PS1 and five FAD mutants in the Aβ peptide segment of the APP revealed that all mutations were associated with decreased γ-secretase activity and a reduced age of disease onset and death [6]. Furthermore, an increase in the ratio between Aβ expression and γ-secretase activity was an early sign of disease in both sporadic and familial AD [6]. The PS2 K115Efx10 mutation causes PS2 protein truncation, and resembles a PS2 isoform, PS2V, which is found in late onset AD brains [7]. Additionally, PS2V mutants were able to activate γ-secretase activity which, under hypoxic conditions, correlated with an attenuation of the unfolded protein response [7]. 

Mature PS1 has many distinct conformational states while non-mature PS1 has only one state [8]. Structural studies of PS1 reveal a so-called “gate-plug” structure where the site responsible for endo-proteolytic cleavage is found. Transmembrane 5 and 6 regions (TM5 and TM6) make up the gate while the exon 9 loop region of the protein makes up the plug. A so-called “unplugging mechanism” by endo-proteolysis and subsequent removal of exon 9 loop is associated with the mature PS1, and susceptibility of a gate-plug region to conformational changes may indicate how PS1 mutants initiate disease [8]. Diminished access and inaccurate cleavage of substrate, along with the altered gate-plug activity, may explain why PS1 mutations are correlated with reduced Aβ levels and increase in Aβ_42_:Aβ_40_ ratio [8]. Changes to TM5 and TM6 histidines (H171A and H197A) reduce self-cleavage of PS1 and interaction with additional γ-secretase constituents, leading to reduced Aβ generation [9]. 

Substitution of histidines with lysine residues in TM5 and TM6 yields structurally normal γ-secretase complexes however with defective enzymatic activity [9]. 

Saturation of γ-secretase with substrate may mechanistically underlie AD pathogenesis by increasing the Aβ_42_:Aβ_40_ ratio, suggesting that competitive γ-secretase inhibitors may be potential therapeutics for AD [10]. Noncompetitive inhibitors, on the other hand, may worsen AD by promoting APP saturation [10]. Two conserved AXXAXXXG motifs were identified in PS1 and PS2, and their involvement in γ-secretase complex configuration were found to be involved in the alternation between normal and pathological γ-secretase conformations [11].

Small molecule γ-secretase modulators were investigated as potential therapies for AD by reducing Aβ_42_ while not blocking γ-secretase processing of substrates [12]. Using a photo-affinity probe, E2012-BPyne, that specifically labeled the N-terminus of PS1 within the active γ-secretase, but not the full-length PS1 in the active form, γ-secretase displayed several binding sites with separate functions [12]. 

The subcellular localization of γ-secretase has been investigated as a contributing factor to Aβ production. The protein Retention in Endoplasmic Reticulum 1 (RER1) controls the intracellular trafficking of γ-secretase [13]. While overexpression of RER1 results in decreased localization of γ-secretase to the cell surface and decreased secretion of Aβ secretion, knockdown of RER1 in turn increased both levels of γ-secretase on the cell surface and Aβ secretion [13]. All in all, increased RER1 decreases the mature APP form leading to reduced surface APP accumulation [13]. 

Mice engineered to express wild type or mutant PS1 in the central nervous system (CNS) and HEK293 cells engineered to express PS paralogs revealed γ-secretase interactions with synaptic vesicle complexes and fusion to cellular membranes as well as H+ transporting lysosomal ATPase complex [14]. The peptidase was mainly co-purified with γ-secretase complexes containing PS2 to control Aβ production [14].

The roles of γ-secretase orthologs from other species have provided clues to non-canonical γ-secretase functions. For example, *Dictyostelium discoideum* γ-secretase orthologs can proteolytically process ectopically expressed human APP to yield Aβ peptides (Aβ_40_ and Aβ_42_), but γ-secretase-deficient strains cannot generate Aβ peptides [15]. *Dictyostelium* γ-secretase was also found to be important for phagocytosis and cell fate determination. These data suggest that phagocytosis may require an active γ-secretase in mammalian and *Dictyostelium* cells [15]. 

In AD patients with mutated PS1, Coupland et al. identified a decrease in the DNA methylation of the promoter for the gene encoding microtubule-associated protein tau (MAPT) as a common phenomenon in a specific brain-region of these AD patients [16].

## 3. Presenilins and Ca^2+^ Signaling

Dysfunction in Ca^2+^ signaling can contribute to age-related central nervous system (CNS) decline [17]. Such damage in brain aging, especially in AD, is thought to be the result of numerous micro-injuries such as oxidative damage in synapses and loss of Ca^2+^ homeostasis leading to increased cytosolic Ca^2+^ concentrations [18]. Long term potentiation is reduced following presynaptic (but not postsynaptic) deletion of presenilins mimicking the depletion of ER Ca^2+^ stores by RyR inhibitors [19]. Presynaptic presenilin deficiency also reduced evoked glutamate release, indicating that presenilins play a role in activity-dependent neurotransmitter release and that presynaptic dysfunction represents an early event in AD development [19]. 

Neurons expressing mutant PS1 exhibit an increase in calcineurin activity and inhibition or reversal of this elevated calcineurin activity stabilized GluA1 phosphorylation and improved homeostatic synaptic plasticity [20]. Improvement of homeostatic synaptic plasticity leads to attenuation of AD-related cognitive decline and likewise improvement in learning and memory [20]. A novel γ-secretase modulator (compound-1) reduces Aβ expression thus relieving cognitive dysfunction in Tg2576 APP transgenic mice, a common mouse model of AD [21]. In mice embryogenic fibroblast cells, this inhibitor also plays a role in Ca^2+^ signaling by enhancing long-term potentiation (LTP), an indicator of synaptic strength [21].

Presenilins are regulators of intracellular calcium stores. RyRs and IP_3_Rs, major intracellular Ca^2+^ channels residing in the ER, are regulated by PS proteins. Furthermore, the expression of ER resident Ca^2+^ channels is increased in neurons expressing mutant PS1 [20]. The presenilin–ryanodine receptor (PS–RyR) interaction, where PS1 and PS2 N-termini bind the cytoplasmic face of RyR, regulates channel activity [22] similar to the actions of other AD related proteins binding to the RyR [23]. Investigation of the expression patterns of PS1 and PS2 identified an overall decrease in PS1 level with increase in PS2 level in older mice [24]. 

A PS1 N-terminal fragment (NTF), which lacks four cysteine residues, decreased total RyR-mediated Ca^2+^ release, while a PS2 NTF, which contains four cysteine residues, had no effect [25]. These cysteines were mutated, allowing conversion of PS1 NTF function to PS2 NTF-like function and vice versa, likely based on differential RyR binding [25]. Inactivation of presenilin in the hippocampus has no effect on ER Ca^2+^ concentration, but in the absence of presenilin, RyR levels and function were decreased in the hippocampus [26]. This suggests a connection between presenilin and Ca^2+^ homeostasis via RyR, further supporting the idea that loss of Ca^2+^ homeostasis is an early pathologic injury in AD [26].

The effect of Aβ plaque proximity to disruptions in hippocampal pyramidal neuron Ca^2+^ signaling was investigated. No significant correlation between Aβ plaque proximity to cells with altered Ca^2+^ signaling was found [27]. These data suggest that early disruptions in pyramidal cell Ca^2+^ signaling occur through Aβ plaque-independent mechanisms [27]. Neuronal presenilins in *Drosophila* have no role on resting Ca^2+^ channels but cause deficits in intracellular Ca^2+^ stores [28]. In addition, calmodulin null mutations suppress presenilin-induced deficits in Ca^2+^ stores [28].

Lee et al., 2015, studied the notion that the mechanism by which PS1 deletion impacts AD was through lysosomal acidification [29]. Their studies revealed that an increased pH in the lysosomes of PS1 knockout (PS1KO) cells caused abnormal Ca^2+^ efflux from lysosomes, resulting in increased cytosolic Ca^2+^ concentrations [29]. Normalizing lysosomal pH restored Ca^2+^ homeostasis, but restored Ca^2+^ homeostasis in turn by itself did not result in adequate acidification of lysosomes or reverse proteolytic and autophagic effects. This led the authors to conclude that an instable lysosomal vesicular ATPase (vATPase) subunit in PS1-deficient cells causes the deficits in lysosomal autophagy [29].

## 4. Presenilins and Oxidative Stress

Oxidative stress is a contributing factor to Alzheimer Disease pathogenesis, with several theories supporting a connection between oxidative stress and the accumulation of Aβ [30]. As monomeric Aβ facilitates glutathione release from astrocytes, it potentially contributes to protection from oxidative stress, a function that is reduced with Aβ_42_ aggregation and subsequent depletion of monomeric Aβ_42_ [31]. Presenilins are involved in neuroprotection against oxidative stress [30]. PS1 was determined to be important for neurotrophic factor-mediated neuroprotection against excitotoxicity and oxidative stress and was not dependent on the role of PS1 in γ-secretase activity, as γ-secretase inhibitors lacked any effect on trypsin-induced neuroprotection [32]. This mechanism seems to stem from PS1 mutants being unable to use trypsin to subsequently rescue neurons from excitotoxicity by activating extracellular signal-regulated kinase 1/2 (ERK1/2) [32]. As expected, PS mutants inhibited neuronal protection against toxic insults [32]. Exposure of neurons to low concentrations (0.25 ppm) of ozone lead to significant increases in Aβ_42_ in mitochondrial fractions, reduction in Aβ_40_, overexpression of PS2, and reductions in ADAM10 expression [30], suggesting that Aβ_42_ accumulation may be involved in mitochondrial dysfunction and subsequent oxidative stress [30]. Sarasija et al. also studied Ca^2+^ transfer, but instead investigated a presenilin analog SEL-12 which regulates ER Ca^2+^ release, demonstrating that mutations in SEL-12 causes mitochondrial fragmentation and dysfunction [33]. This role in mitochondrial damage did not require γ-secretase activity and amyloid plaques [33]. 

The effect of certain diabetes drugs on Aβ production and oxidative stress has been investigated. Administration of the insulin sensitizer, metformin, increases APP and presenilin expression via NF-κB activation [34]. In contrast, insulin administration antagonized the effects of metformin by decreasing Aβ levels and reducing oxidative stress and mitochondrial dysfunction [34]. Interestingly, monomeric Aβ_42_ is capable of activating the phosphatidylinositol-3-kinase pathway and thereby generates neuroprotection via insulin-like growth factor-1 and other receptors [35]. This raises the interesting notion that part of Aβ toxicity is the result of a depletion of Aβ_42_ subsequent to Aβ_42_ oligomerization and polymerization [35].

The relationship between mitochondrial function and chaperone-mediated RyR degradation in cardiomyocytes (as well as fibroblast number) was studied in AD patients with PS1 mutations [35]. Fibroblasts with the AD mutation had elevated Aβ_42_, reduced ATP levels, reduced mitochondrial respiration, and impaired mitochondrial respiratory capacity [36]. 

Copper (Cu^2+^) is important for enzymatic antioxidant activity, namely as a cofactor in the antioxidant enzyme superoxide dismutase (SOD) [37]. While PS1 and PS2 play roles in Cu^2+^ uptake, presenilin knockdown in *Drosophila* reduces Cu^2+^ levels and consequently decreases SOD [37]. These presenilin knockdown *Drosophila* were sensitive to SOD-inducing chemical paraquat, supporting the role of presenilin on SOD activity [37]. Interestingly, in Zebrafish, a truncated PS2 isoform, PSV2, is induced in spontaneous AD under hypoxic conditions and conditions of high cholesterol [38]. PSV2 normally increases γ-secretase activity [38]. Zebrafish possess another presenilin isoform, PS1IV, an isoform similar to PS2V in humans [38]. It is associated with changes in cytokine gene expression, such as IL1β and CCR5, and in addition, the absence of PS1IV under hypoxic conditions is associated with changes in vascular development, protein synthesis, Ca^2+^ homeostasis, and cell proliferation [38].

*Drosophila* presenilin interacts with the enzymes thiol-specific antioxidant (TSA) and proliferation-associated gene (PAG), both involved in cellular antioxidant activity, and thereby affects Notch signaling [39]. Transgenic presenilin expression in precursor cells of wing and sensory organ caused a Notch loss-of-function phenotype [38]. In fact, co-expression of presenilin with proteins resulted in a more severe and penetrant Notch loss-of-function phenotype than PS expression alone [39]. Such signaling mechanisms involved in inflammation appear to be of particular importance given the role inflammation has in AD development in the presence of high Aβ levels [40] and that other pathogenic signaling mechanism such as tau protein cleavage and of the formation of neurofibrillary tangles respond to intervention with antioxidants [41]. 

Pedrozo et al. induced chaperone-mediated autophagy (CMA) in cardiomyocytes with geldanamycin, which prevented the loss of RyR2 degradation, suggesting that presenilins were involved in this process [42]. Presenilins, therefore, are involved in CMA and can target oxidatively damaged RyR2 [42].

## 5. The Role of Presenilins in Proteasome Function and Autophagy

Presenilin has many roles including, but not limited to, RyR regulation and interaction with other regulatory pathways. Hwang et al. demonstrated that PS2 mutations can lead to NF-κB mediated amyloidosis [43]. Presenilins have two roles: proteolysis-dependent activity in the γ-secretase complex and activities in cellular signaling that are independent of proteolytic activity [44]. The coupling of ubiquitin conjugation to endoplasmic reticulum degradation (CUE) ubiquitin binding domain of PS1 coordinates polyubiquitination at lysine 63 [45]. 

Recent studies determined the effect of presenilins in the autophagy/lysosome system and found that presenilin deficit led to a reduction in lysosomal Ca^2+^ stores regardless of lysosome accumulation, and prevention of the organization of two-pore channels 1 and 2 (TPC1 and TPC2) [46]. This indicates that modifications in lysosomal Ca^2+^ due to presenilin deficiency can lead to interference of autophagy [46]. In addition, genetic deletion or knockdown of presenilins can lead to a buildup of autophagosomes independent of γ-secretase activity [47]. Ablation of *Dictyostelium* presenilins lead to PS1-mediated restoration of the terminal differentiation of multiple cell types independent of its proteolytic effect [44]. Presenilin loss in *Dictyostelium* leads to elevated cAMP concentrations and elevated Ca^2+^ release, indicating that presenilins indeed regulate signaling pathways [44]. 

The impact of loss of PS1 activity on lysosomal alkalization and subsequent impairment of autophagosomal function was determined, but investigations were unable to identify presenilin involvement in controlling autophagy [48]. Studies of mice brains lacking PS, however, revealed a function for PS in regulating lysosomal formation [48].

*Tequila* and mammalian analog *Prss12* gene expression is reduced by presenilins in brains of *Drosophila melanogaster* larvae and in mouse embryonic fibroblasts [49]. A mature γ-secretase complex was found to be essential for inhibiting neurotrypsin expression and reduction of agrin cleavage, but PS1 processing of γ-secretase substrates was not required for this activity [49]. Silencing of the *Drosophila* ortholog of presenilins (dPsn) lowered the heart rate, while dPsn overexpression increased it [50]. dPSN silencing also increased dIP_3_R expression and decreased dSERCA expression, while dPsn overexpression lowered dRyR expression [50]. All in all, changes in presenilin expression resulted in cardiac dysfunction via aberrant Ca^2+^ signaling and disrupted Wnt signaling [50] (summarized in Table 1).

## 6. Functions of Presenilins Outside of AD

Besides its well-documented role in AD, presenilins also play many roles in other diseases (see Table 2). This results in both a more differentiated view of the involvement of PS and potentially opens up new avenues for drug targeting and drug discovery. The role of a gene, which interacts with PTEN-induced putative kinase in mitochondrial homeostasis and during early-onset Parkinson disease, called presenilin-associated rhomboid-like (*PARL*), was investigated [51]. Single nucleotide polymorphisms in PARL represented a rare cause of Parkinson disease [51]. 

Presenilin is also involved in variants of cancer, as PS1 was amplified in gastric cancer and correlated with a poor survival and increased metastasis [52]. This mechanism may be explained by the E-cadherin cleavage and β-catenin release by PS1, thus allowing β-catenin nuclear translocation and transcriptional activations to promote gastric cancer progression [52]. Fusion transcripts between large tumor suppressor 1 (*LATS1*) and PS1 genes were unable to phosphorylate yes-associated protein and subsequently inhibit the growth of malignant mesothelioma cells [53]. 

PS1 is also involved in the development of the skin disorder hidradenitis suppurativa or acne inversa. Defective Notch signaling due to loss of function mutations of PS-1 and other γ-secretase subunits likely contributes to the pathogenesis of hidradenitis suppurativa affecting integral membrane proteins such as Notch, E-cadherin, or CD44 [54]. A Mutation of PS2 was identified as a genetic cause for familial comedones syndrome, which has clinical phenotypes similar to hidradentis suppurativa [55]. 

While a clear link has been demonstrated between development of Alzheimer’s disease and increasing age, links have also been found between PS function and normal aging. A preclinical model for aging was used to identify changes in cerebellar and forebrain PS expression that correlate with performance in motor function, memory, and learning in aged rats, where PS1 was decreased while PS2 was increased [24]. Puig et al. identified the roles of mutant APP and PS1 in the enteric nervous system [56]. They found that APP/PS1 mice had normal gastrointestinal function, but they had higher luminal IgA and APP, indicating elevated proinflammatory factors and immune cell activation [56]. 

Presenilins also play a role in cardiac function. Chaperone-mediated autophagy (CMA), a process involved in the degradation of soluble proteins in the cytosol, occurs by lysosome associated membrane protein type 2A- (LAMP-2A)-facilitated degradation [42]. LAMP2 mutations can lead to Danon disease, characterized by hypertrophic cardiomyopathy [42]. Pedrozo et al. discovered that RyR2 is degraded by CMA, suggesting that oxidative damage targets RyR2 for turnover via presenilins and CMA [42]. Li et al. discovered that silencing the *Drosophila* ortholog of presenilins (dPsn) reduced heart rates and generated an age-dependent rise in end-diastolic vertical dimensions; conversely, dPsn overexpression led to higher heart rates [50]. Silencing of dPsn elevated the expression levels of the *Drosophila* ortholog of IP_3_R and reduced expression of the *Drosophila* ortholog of SERCA while overexpression of dPsn led to reduced expression of the *Drosophila* ortholog of the RyR [50], offering a mechanism for how cardiac dysfunction occurs via changes in PS expression. Overall, presenilin changes lead to cardiac dysfunction secondary to abnormal Ca^2+^ channel activity and disrupted Wnt signaling [50]. Presenilins also play a role in embryogenesis. Donoviel et al. generated PS1/PS2 double null mice and noticed embryonic lethality [57]. In addition, embryos deficient in both presenilins demonstrated developmental dysregulation such as absence of segmentation, defects in ventral neural tube formation, delays in the closure of the anterior neuropore, and irregular heart development [57].

## 7. Conclusions

Overall, the involvement of PS as part of the γ-secretase complex and in other roles in both excitable and non-excitable cells, but especially in immune cells such as T-cells and macrophages (Table 1), opens up a wide range of possible roles for PS as targets for AD drug target discovery and drug development (Table 2).

## Figures and Tables

**Table 1:** Presenilin function within cells.

Presenilin Function	Protein/Signaling Targets	References
γ-secretase complex activity	APP	[2,6,7,10,11,12,13,14,15]
Ca^2+^ signaling	IP_3_R, RyR (mammalian); regulation of dIP_3_R, dSERCA and dRyR expression (*Drosophila melanogaster*); SEL-12 (*Caenorhabditis elegans*)	[4,17,18,19,20,21,22,25,26,28,29,33,46]
Oxidative stress	trypsin-mediated ERK1/2 activation, mitochondrial proteins, thiol-specific antioxidant (TSA) and proliferation-associated gene (PAG)	[18,30,32,39,42]
Proteolysis	Trypsin, CREB activity	[32,49]
Lysosome/Autophagy	vATPase regulation, chaperone-mediated autophagy, two-pore calcium channel expression, lysosomal proteolysis, lysosomal acidification	[29,42,46,47,48]
Cellular signaling	Notch, inflammatory signaling	[38,39]
Cu^2+^ uptake	reduced Cu^2+^ uptake, reduced SOD expression	[37]
Cellular differentiation/development	Proteolytic agrin cleavage	[15,44,49]

**Table 2:** Presenilin involvement in diseases and conditions.

Disease/Condition	System/Organ	References
Normal neuronal function (cognition, memory)	Brain, intestine	[19,21,24,26,28,32,42,43]
Alzheimer’s disease	Brain	[1,4,5,6,16,17,19]
Parkinson’s disease	Brain	[51]
Familial comedones	Skin	[54,55]
Cancer	gastrointestinal	[52,53]
Cardiac dysfunction (embryonic development)	heart	[42,50,57]

## Abbreviations

Aβ	amyloid beta
AD	Alzheimer’s disease
APP	amyloid precursor protein
CMA	chaperone-mediated autophagy
CNS	central nervous system
ER	endoplasmic reticulum
ERK1/2	extracellular signal-regulated kinase 1/2
FAD	familial Alzheimer’s disease
IP_3_R	inositol 1,4,5-trisphosphate receptor
LAMP	by lysosome associated membrane protein
LATS1	large tumor suppressor 1
MAPT	microtubule-associated protein tau
NTF	N-terminal fragment
PS1	presenilin 1
PS2	presenilin 2
RyR	ryanodine receptor
SOD	superoxide dismutase
TPC	two-pore channels
